# Supplementary material for: Development of 3D ZnO-CNT Support Structures Impregnated with Inorganic Salts
Source: Membranes (Basel). 2022 May 31;12(6):588. doi: 10.3390/membranes12060588 (PMC9229228; doi:10.3390/membranes12060588)
Supplement: Supplementary file 1 [file membranes-12-00588-s001.zip › membranes-1736285-supplementary.pdf]

membranes

Development of 3D ZnO-CNT support structures impregnated with inorganic salts

Stefania Chiriac[1+], Maria-Eliza Puscasu[1+], Ioan Albert Tudor[1], Alexandru Cristian Matei[1], Laura Madalina Cursaru[1,] * and Radu Robert Piticescu [1,] *

* Correspondence: mpopescu@imnr.ro (L.M.C) ; rpiticescu@imnr.ro (R.R.P.).

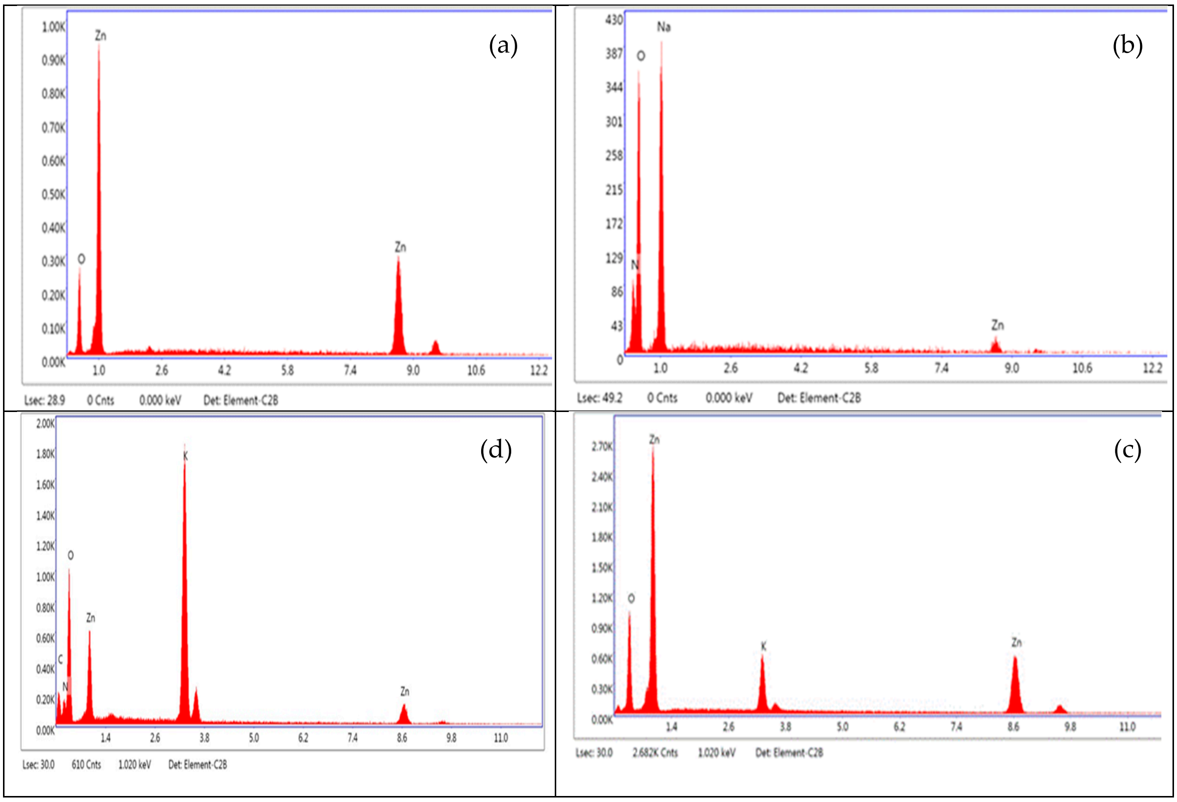

Figure S1. SEM-EDS spectra of the 3D suport structures, before impregnation (a) andafter impregnation with $NaNO_3$ (b), KNO_3 (c) and $NaNO_3$: KNO_3=1:1 (d)

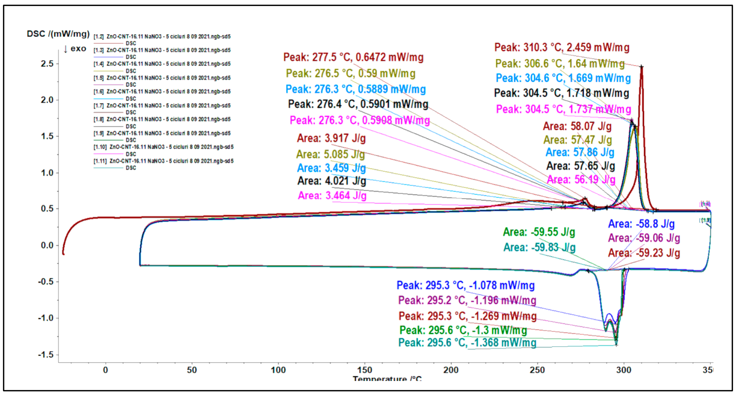

Figure S2. DSC results of $NaNO_3$ impregnated 3D structures

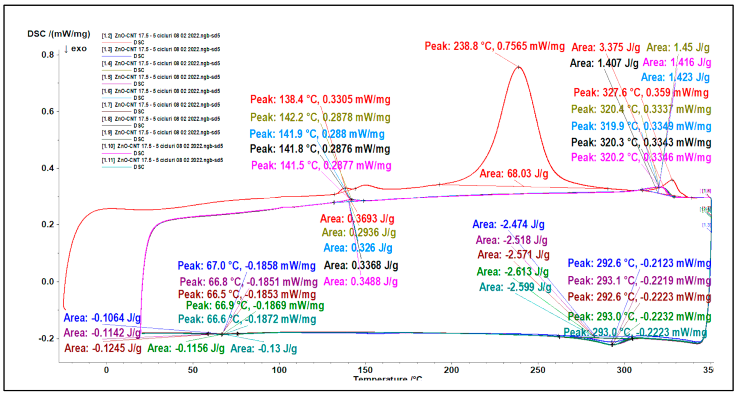

Figure S3. DSC results of KNO_3 impregnated 3D structures

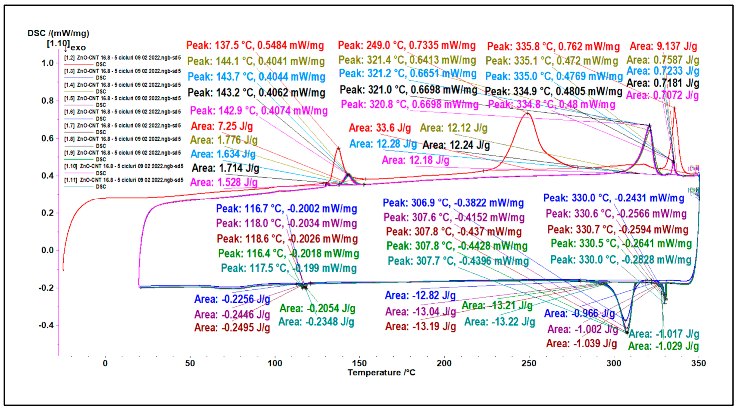

Figure S4 DSC results of $NaNO_3$ – KNO_3 impregnated 3D structures
